# Inter-Individual Variation during Transcranial Direct Current Stimulation and Normalization of Dose Using MRI-Derived Computational Models

**DOI:** 10.3389/fpsyt.2012.00091

**Published:** 2012-10-22

**Authors:** Abhishek Datta, Dennis Truong, Preet Minhas, Lucas C. Parra, Marom Bikson

**Affiliations:** ^1^Neural Engineering Laboratory, Department of Biomedical Engineering, The City College of City University of New YorkNew York, NY, USA; ^2^Soterix MedicalNew York, NY, USA

**Keywords:** tDCS, head model, HD-tDCS, TMS, tACS, transcranial electrical stimulation

## Abstract

**Background:** Transcranial Direct Current Stimulation (tDCS) is a non-invasive, versatile, and safe neuromodulation technology under investigation for the treatment of neuropsychiatric disorders, adjunct to rehabilitation, and cognitive enhancement in healthy adults. Despite promising results, there is variability in responsiveness. One potential source of variability is the intensity of current delivered to the brain which is a function of both the operator controlled tDCS dose (electrode montage and total applied current) and subject specific anatomy. We are interested in both the scale of this variability across anatomical typical adults and methods to normalize inter-individual variation by customizing tDCS dose. Computational FEM simulations are a standard technique to predict brain current flow during tDCS and can be based on subject specific anatomical MRI. **Objective:** To investigate this variability, we modeled multiple tDCS montages across three adults (ages 34–41, one female). **Results:** Conventional pad stimulation led to diffuse modulation with maximum current flow between the pads across all subjects. There was high current flow directly under the pad for one subject while the location of peak induced cortical current flow was variable. The High-Definition tDCS montage led to current flow restricted to within the ring perimeter across all subjects. The current flow profile across all subjects and montages was influenced by details in cortical gyri/sulci. **Conclusion:** This data suggests that subject specific modeling can facilitate consistent and more efficacious tDCS.

## Introduction

Transcranial Direct Current Stimulation (tDCS) has gained widespread popularity for being a non-invasive, cheap, safe therapy investigated for treating a host of neurological disorders, enhancing cognitive abilities, and as an adjuvant rehabilitation treatment (Nitsche and Paulus, 2000; Antal et al., 2004; Fregni et al., 2006; Edwards et al., 2009; Baker et al., 2010; Loo et al., 2012). During tDCS, the current injected through scalp electrodes induces electric fields (EF) in the cortex which is believed in turn to modulate neuronal excitability (Nitsche and Paulus, 2000). This modulation of membrane excitability ultimately determines observed behavioral/clinical outcomes.

Since its introduction in its current form (Nitsche and Paulus, 2000), there is still limited knowledge of how to optimally determine treatment “dose” – where dose is defined by electrode placement/size or stimulus parameters (current intensity, polarity, session duration) controllable by the operator (Bikson et al., 2008; Peterchev et al., 2011). While, these various dose options underlie the inherent flexibility of tDCS, they also make the optimal choice difficult to ascertain (Brunoni et al., 2012). It is reasonable to assume that cortical regions subject to higher current flow intensities are more likely candidates for modulation and plasticity. Importantly, the distribution of current flow in the brain depends not only on the stimulation dose but underlying anatomy/tissue properties. In this way, the same dose applied to two subjects may result in different brain current flow patterns (Chaieb et al., 2008). Furthermore, the same dose across healthy subjects and subjects with compromised anatomy (lesions, skull defects) may lead to varied brain regions activated by current flow and thus inconsistent clinical outcomes.

It is known that there are age-related anatomical differences spanning the pediatric to the elderly population. Even within a particular age group, there is remarkable inter-individual variability in anatomy both at the level of whole tissue volume/thickness and cortical morphology. For example, brain volume across 30 individuals aged (18–35) was found to vary by as much as 40% (Song et al., 2011). Cortical gyri-sulci morphology (contours, folding patterns, functional localization) are complex and are characterized by high inter-individual variability (Mangin et al., 2004; Derrfuss et al., 2009). This is of particular significance since the gyrated structure of the brain has been implicated in the observance of current “hot-spots” in high-resolution modeling (see supplementary figure – Datta et al., 2009). Furthermore, studies have suggested gender-related differences: (1) males have higher CSF and white matter volume while females have higher gray matter volume (Gur et al., 2002) and (2) females might have thicker skulls than men. It remains to be seen whether these aforementioned differences may translate to a significant difference in tDCS current flow patterns across individuals.

Computational modeling using finite element (FE) methods is an established tool for predicting tDCS current flow and thus should be leveraged to plan dosing strategies. Recent studies have attempted to directly compare modeling predictions to clinical outcomes thereby validating the utility of this approach (Mendonca et al., 2011; Dasilva et al., 2012; Turkeltaub et al., 2012). In addition, we have recently used patient-specific modeling for tDCS responders to: (a) retrospectively analyze the success of a given montage in aphasia stroke (Datta et al., 2011) and (b) compared model predictions to physiological patterns of activation revealed by fMRI in visual stroke (Halko et al., 2011).

Transcranial direct current stimulation studies are usually planned by assuming increased/decreased excitability “under” the anode/cathode electrode respectively or by placing the active electrode “over” the desired region-of-interest with the return electrode placed on a distant location – contralateral hemisphere or at extra cephalic locations. The increased proliferation of studies over the last decade has shown that this heuristic strategy has proven efficacious. But this simple approach is not consistent with imaging/modeling studies which suggest broad neuronal activation with peak brain modulation potentially between electrodes (Lang et al., 2005; Datta et al., 2009; Sadleir et al., 2010; Salvador et al., 2010). One source of observed variability across subjects could therefore be variation in the location of peak brain current flow as well as overall current flow patterns.

As a first step toward considering the impact of anatomical differences in resulting brain current flow across healthy adults, we modeled tDCS induced electrical fields in three adults: two males (M1, M2) and one female (F) via high spatial resolution (1 mm^3^) gyri-sulci precise computer modeling. The magnitude and the spatial extent of conventional sponge-pad and High-Definition (HD)-tDCS were compared across subjects (Datta et al., 2009; Borckardt et al., 2012). HD montages allow focal delivery of current to select regions of the cortex. We report that tDCS modulation maps may be fundamentally influenced by the underlying individual head anatomy.

## Materials and Methods

**Figure 1:**
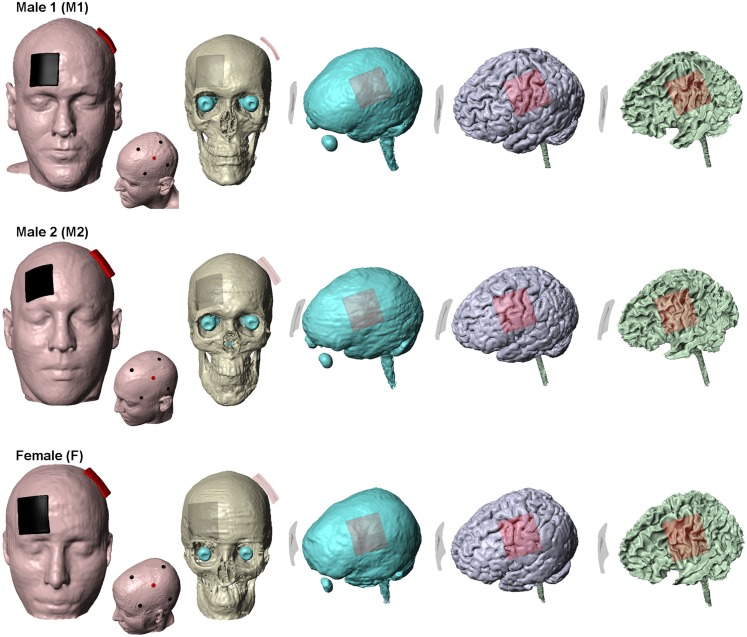
**Segmentation masks**. Subject specific tissue masks of the three subjects used in the study. Skin, skull, CSF, gray matter, and white matter are shown. The traditional sponge and the 4 × 1-HD montage for each of the subjects are also shown. For the sponge montage, the anode (positive) electrode is placed over left M1 and the cathode (negative) electrode over the contralateral-supraorbita. For the 4 × 1-HD montage, the anode electrode is placed over left M1 and is surrounded by four cathode electrodes. Red: anode (positive) electrode and Black: cathode (negative) electrode.

We obtained T1 and T2 scans at 1 mm^3^ resolution from three healthy neurologically normal subjects: Male 1 (M1): 36 years; Male 2 (M2): 41 years and Female (F): 34 years. Automated segmentation was first performed using SPM (Ashburner, 2009) to demarcate the MRI images into six tissue categories: skin, skull, CSF, gray matter, white matter, and air. An in-house MATLAB script (Huang et al., 2012) was used to correct for the automatic segmentation errors. Residual segmentation errors were finally fixed in ScanIP (Simpleware, Ltd., Exeter, UK) using a combination of segmentation tools (point to point line, smoothing filters, and Boolean operations). The stimulation electrodes were created as CAD files and were positioned interactively within the image data (Figure 1). Adaptive FE meshes were generated with a minimum quality factor of 0.4 from the segmentation and the CAD masks (Simpleware). The entire workflow preserved the resolution of the anatomical 1 mm resolution data (Bikson and Datta, 2012). The meshes were imported to COMSOL Multiphysics 3.5a (Burlington, MA, USA) for FE computation and comprised >10 million elements with >15 million degrees of freedom. Electrical conductivities (S/m) were assigned the representative average values obtained from literature: Skin (0.465); Skull (0.01); CSF (1.65); Gray matter (0.276); White matter (0.126); air (1e−7); electrode (5.8e7); sponge (1.4); gel (0.3) (Wagner et al., 2007).

The two modeled electrode configurations for each of the three heads were as follows:

(1) Conventional “rectangular-pad”– Two electrode-sponge pads (5 cm × 5 cm) were placed at sites commonly used for the classic motor cortex-contralateral orbital stimulation (Figure 1). Typically sponges are soaked in saline for tDCS application – sponges were thus assigned saline’s conductivity and the abutting electrode energized.
(2) HD 4 × 1-ring – Four cathode disk electrodes were arranged in a circular fashion around an anode center electrode (Datta et al., 2009; Borckardt et al., 2012). The anode electrode is placed over the motor cortex coinciding with the center of the anode pad used for conventional stimulation (Figure 1). All electrodes had a diameter of 12 mm and an electrode-center to electrode-center distance of 6 cm from the central anode electrode was used. Current was conducted into the head via a gel.

**Figure 2:**
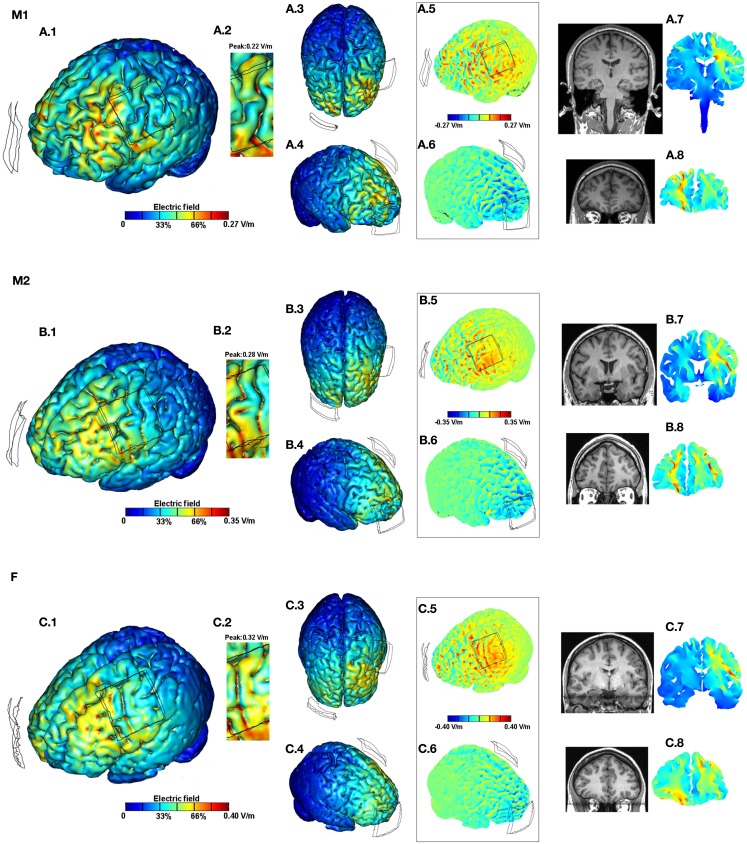
**Brain modulation across subjects (M1, M2, F) using conventional pad configuration**. For each subject we plotted the induced cortical *surface* electric field (EF) magnitude: left side view **(A.1,B.1,C.1)**; top view **(A.3,B.3,C.3)**; and right side view **(A.4,B.4,C.4)**. The motor cortex is expanded and scaled to 80% of the peak induced EF for each of the subjects to better highlight current flow **(A.2,B.2,C.2)**. The boxed images show the directional plots **(A.5,A.6,B.5,B.6,C.5,C.6)**. Sample *cross-section* EF magnitude plots were taken for the frontal and the motor regions. The corresponding MRI scan collected for the subject and the cross-section plots are shown juxtaposed to each other **(A.7,A.8,B.7,B.8,C.7,C.8)**.

**Figure 3:**
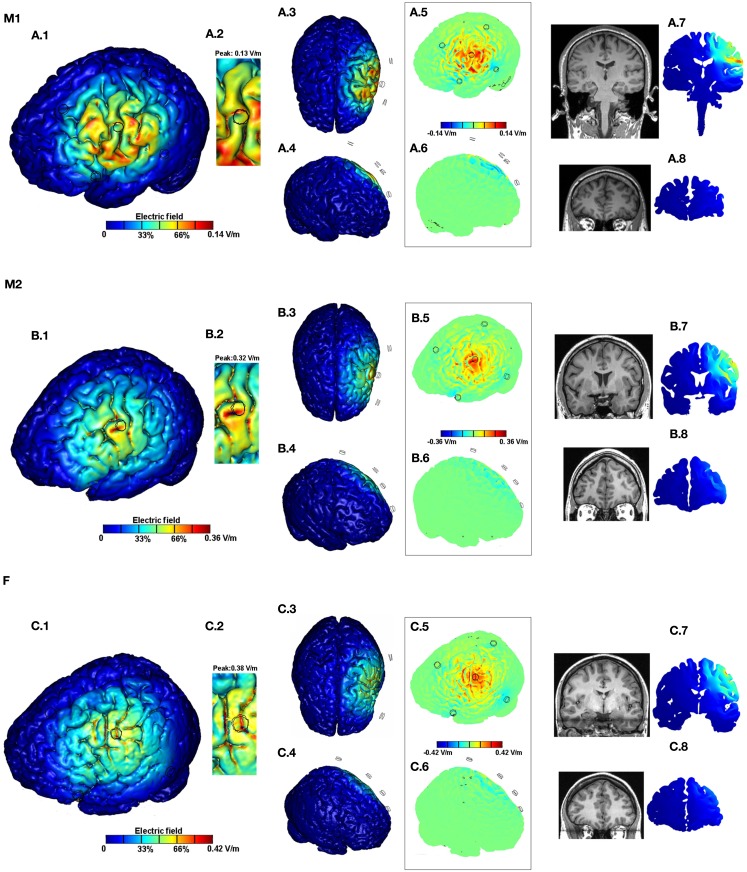
**Brain modulation across subjects (M1, M2, F) using high-definition 4 × 1 configuration**. For each subject we plotted the induced cortical *surface* electric field (EF) magnitude: left side view **(A.1,B.1,C.1)**; top view **(A.3,B.3,C.3)**; and right side view **(A.4,B.4,C.4)**. The motor cortex is expanded and scaled to 90% of the peak induced EF for each of the subjects to better highlight current flow **(A.2,B.2,C.2)**. The boxed images show the directional plots **(A.5,A.6,B.5,B.6,C.5,C.6)**. Sample *cross-section* EF magnitude plots were taken for the frontal and the motor regions. The corresponding MRI scan collected for the subject and the cross-section plots are shown juxtaposed to each other **(A.7,A.8,B.7,B.8,C.7,C.8)**.

The standard Laplace equation was solved using conjugate gradients iterative solver with a tolerance of 1 × 10^−6^. 1 mA total current was applied at the anode electrode and ground was applied at the negative electrode(s). The remaining external surfaces were considered as insulated. Cortical EF surface and cross-section magnitude maps were determined (Figures 2 and 3). The surface EF magnitude maps were plotted to the respective induced peak on the cortical surface. In addition, directional plots normal to the cortical surface (inward or outward) were plotted (Datta et al., 2008; Turkeltaub et al., 2012).

## Results

For the conventional 5 × 5 pad tDCS and the 4 × 1-ring HD-tDCS configurations, we calculated induced cortical EF across all subjects. The surface/cross-section magnitude plots for each combination (montage and subject) allow a direct comparison of the spatial profile and depth focality. In addition, the role of inter-individual differences is further demonstrated by the consideration of current flow direction and zoomed views of a region-of-interest (motor strip). Barring the zoomed views, each of the false-color plots have been plotted to the respective peak EF induced on the cortical surface.

### Conventional pad stimulation

Conventional pad stimulation resulted in current clustering with diffuse modulation over wide parts of the cortex (Figures 2A.1, B.1,C.1). The top view (Figures 2A.3,B.3,C.3) together with the right side view (Figures 2A.4,B.4,C.4) further highlight the widespread nature of current flow across the entire cortical surface. This is attributable to the large size/separation of the pads and gyrated anatomy. Consistent with previous predictions, the overall current flow was complex, reflecting the convoluted gyri-sulci morphology and individual neuroanatomy (Datta et al., 2009, 2011; Salvador et al., 2010). tDCS across subjects resulted in distinct predicted EF distributions in the brain. Maximal current flow was generally induced in the frontal regions *between* the electrodes across all subjects. While subject F resulted in relatively higher current flow directly *underneath* the C3 pad, the motor strip is largely spared for M1. A total current of 1 mA injected through the electrodes resulted in 0.27, 0.35, and 0.40 V/m peak cortical EF magnitudes for M1, M2, and F, respectively. Thus there is a ∼1.5-fold variation in the predicted peak induced EF values across the three anatomically normal adult subjects.

Though global individual variation in peaks and clustering is apparent by inspection, the importance of detailed and individual anatomy is further highlighted by the consideration of the zoomed regions. The zoomed motor regions have been re-plotted to 80% of the respective peak EF induced for each of the subjects to better highlight regional current flow (Figures 2A.2,B.2,C.2). On both macro- and micro-scales, both peak and relative current flow patterns are subject specific using the identical tDCS montage.

The boxed images showing the directional EF normal to the cortical surface distinguishes current flow direction (Figures 2A.5,A.6,B.5,B.6,C.5,C.6) where inward/outward direct current is expected to produce somatic depolarization/hyperpolarization (Radman et al., 2009). Here again, differences in both the peak and pattern of current flow are apparent. Finally, the sample coronal cross-section plots (taken through the motor and the frontal regions) confirm the diffuse bilateral nature of current flow with the pad montage and individual variation in patterns across deep brain structures in both the frontal (Figures 2A.8,B.8,C.8) and motor cross-sections (Figures 2A.7,B.7,C.7). Subject specific local peaks are observed across the cross-sections, presumably reflecting anatomical idiosyncrasies such as proximity to ventricles.

### HD stimulation

For all subjects, 4 × 1-ring HD-tDCS montage resulted in cortical activation circumscribed by the ring thereby leading to significant focality increases (Figures 3A.1,B.1,C.1). There was no significant current flow modulation in the frontal, contralateral, or on the occipital side of the brain as evidenced by the top (Figures 3A.3,B.3,C.3) and the right side views (Figures 3A.4,B.4,C.4). A total current of 1 mA injected through the electrodes resulted in 0.14, 0.36, and 0.42 V/m peak cortical EF magnitudes for M1, M2, and F, respectively. Thus there is a ∼3-fold increase in the induced EF values going from M1 to F. Inspection of global current patterns within the ring, as well as detailed consideration of the motor strip (Figures 3A.2,B.2,C.2; re-plotted to 90% of the respective peak EF) indicates idiosyncratic variations within the ring including difference in the rate of peak EF drop off, moving away from the center electrode. The boxed directional images confirm the unidirectional nature of the 4 × 1 montage of previous studies (Datta et al., 2008) – inward current is mostly restricted to within the cortical regions directly underneath the center electrode and the outward current is diffuse (Figures 3A.5,A.6,B.5,B.6,C.5,C.6). The cross-section plots confirm no modulation in the frontal regions and contralateral motor regions for all subjects (Figures 2A.7,A.8,B.7,B.8,C.7,C.8) with moderate variation in depth penetration across subjects.

## Discussion

In this study, three high-resolution anatomically accurate head models were studied to investigate the variations in current flow patterns (spatial profile/peak) due to conventional and HD montages. The observance of distinct localized clusters/hot-spots across healthy subjects reinforces the need to incorporate detailed cortical anatomy in determining brain current flow. Additionally, the variation in global patterns and the peak cortical current flow across subjects highlights the need of individual anatomy.

As expected, conventional montage was characterized by un-focal diffuse current flow while the HD montage led to field distributions restricted to within the outer ring perimeter consistent with previous modeling efforts (Datta et al., 2009; Suh et al., 2010). It follows that the diffuse current flow produced during conventional pad tDCS *aggravates* individual differences. tDCS resulted in several peak clusters spanning the frontal lobe including cortical and deeper structures. Though for these three subjects, the peak EF varied more for 4 × 1-ring HD-tDCS compared to conventional tDCS (3× vs. 1.5×), the peak EF remained confined to the cortex under the center electrodes and in no case did current invade brain regions substantially outside the ring. The maximum EF on localized hotspots at the bottom of the sulci may have contributed to a bigger variation for the 4 × 1 montage. Furthermore, it has been previously reported that 2 mA – 4 × 1 at 3 cm separation corresponds to comparable EFs at 1 mA sponge stimulation. The results of this study show that at 6 cm separation – 1 mA, 4 × 1 may lead to comparable or even higher EFs in comparison to sponges.

The viability of HD stimulation was first shown in the Minhas et al. (2010) study by using appropriate hardware (electrode material, gel, and electrode adapters). Since then several clinical studies have been initiated in healthy and diseased subjects to explore the efficacy of HD-tDCS stimulation. 4 × 1-Ring HD-tDCS has been shown to be efficacious for experimental pain (Borckardt et al., 2012) and in Transcranial Magnetic Stimulation – Motor Evoked Potential (Caparelli-Daquer et al., 2012) studies. While these initial studies additionally address the viability of this technique and its safety/tolerability profile, they do not directly address whether a more targeted therapy equates to a more beneficial outcome. Naturally, future research will have to adjudicate whether a focal therapy will lead to similar, worse, or better outcomes than traditional sponge electrode montages.

It is not tractable to explicitly compare brain current flow across hundreds of heads using currently available computational resources and software (as usually done by MR analysis to study inter-individual anatomical differences; Gur et al., 2002). Rather the goal on this study was to access the degree of potential changes expected even across comparable age healthy adults. One may venture into general dose guidelines, such as the role of head-size, gender, or if the order of EF sensitivity will be maintained across montages, but with this limited set of data, this is speculative. Thus further automation of the modeling process remains critical for economical and broad dissemination. Inferences are further complicated, as there is likely no simple (one to one) relationship between current in any given region and behavioral/clinical outcomes. What is clear is that changes in peak brain EF ∼3-fold can be expected and potentially more if more diverse adult healthy individuals are considered. If one assumes that roughly doubling or more stimulation intensity is functionally meaningful (as indeed shown in clinical studies), then these results suggest difference in current flow due to individual differences is a significant source of variability in tDCS.

What steps can be taken to normalize dose? In regards to peak EF, the simplest approach is to “scale” applied current across subjects. For example, stimulation using the M1-SO montage in subject M1 using 1.5 mA produces comparable peak EF as stimulation in subject F. More generally, if the model predicted × times higher current in the target region for a head than for a baseline “efficacious” head, a simple way to “normalize” dose would be reduce the total injected current by a factor of ×. A variation of up to ∼3.7-fold in peak EF was predicted in a study comparing an idealized skull defect to a healthy adult head (Datta et al., 2010). Likewise, higher variation is expected going from pediatric to elderly population. Normalizing dose across a diverse population thus requires subject specific MRI-derived models using available gross anatomical features (such a system is in development at City College New York: CCNY-Dose System). However, normalizing for variation in current flow pattern is more complex and cannot be addressed by simply changing applied currents or adjusting pad placement. In this regard, montages such as the 4 × 1-ring are compelling because they, at a minimum, at least constrain which brain regions are potentially modulated.

Though we expect the main conclusions of this study are robust, the accuracy of any FEM model is limited not only by the precise representation of anatomy but also by material properties (including anisotropy). Preservation of 1 mm resolution throughout the modeling workflow led us to accurately capture individual specific cortical folds/contours, skull architecture, continuous CSF layer – which consequently led to the individual differences, reported here. Improving the precision of the model by incorporating DTI conductivities in the anisotropic (white matter and the skull) regions as well as to establish reliable DC conductivities for the remaining isotropic regions is needed. More importantly, directly validating the patient-specific modeling predictions by their individual functional effects by applying DC stimulation (e.g., MEP changes following motor cortex stimulation) in a clinical study is ideally required.

Keeping with the ultimate goal of optimizing tDCS therapy and reducing variability, consideration of current flow patterns remains paramount for design of montages and interpretation of patient-specific results – thus the ability to individualize therapy must be leveraged. The predictions of this study are the first step to explore reported inter-individual differences via computer modeling. The data suggest that individualized modeling may require consideration in determining tDCS efficacy. Future work will need to determine whether subject specific dosing based on modeling is meaningfully beneficial for tDCS outcomes or if currently used fixed-dose approaches are sufficient.

## Conflict of Interest Statement

Dr. Datta is co-founder of Soterix Medical. The City University of New York has patent applications in Dr. Datta’s name on brain stimulation. The City University of New York has patent applications in Dr. Parra’s name on brain stimulation. Dr. Parra is co-founder of Soterix Medical. The City University of New York has patent applications in Dr. Bikson’s name on brain stimulation. Dr. Bikson is co-founder of Soterix Medical.
